# The Role of Copper Chaperone Atox1 in Coupling Redox Homeostasis to Intracellular Copper Distribution

**DOI:** 10.3390/antiox5030025

**Published:** 2016-07-27

**Authors:** Yuta Hatori, Svetlana Lutsenko

**Affiliations:** 1 Faculty of Pharmacy, Yasuda Women’s University, Hiroshima 731-0153, Japan; hatori@yasuda-u.ac.jp; 2 Department of Physiology, Johns Hopkins University School of Medicine, 725 N. Wolfe street, Baltimore, MD 21205, USA

**Keywords:** copper, heavy metal, glutathione, Atox1, copper chaperone

## Abstract

Human antioxidant protein 1 (Atox1) is a small cytosolic protein with an essential role in copper homeostasis. Atox1 functions as a copper carrier facilitating copper transfer to the secretory pathway. This process is required for activation of copper dependent enzymes involved in neurotransmitter biosynthesis, iron efflux, neovascularization, wound healing, and regulation of blood pressure. Recently, new cellular roles for Atox1 have emerged. Changing levels of Atox1 were shown to modulate response to cancer therapies, contribute to inflammatory response, and protect cells against various oxidative stresses. It has also become apparent that the activity of Atox1 is tightly linked to the cellular redox status. In this review, we summarize biochemical information related to a dual role of Atox1 as a copper chaperone and an antioxidant. We discuss how these two activities could be linked and contribute to establishing the intracellular copper balance and functional identity of cells during differentiation.

## 1. Introduction

Copper is the major redox-active element in most biological systems. As a redox catalyst, copper is utilized in many essential cellular processes including energy production by the respiratory chain, biosynthesis and degradation of neurotransmitters, formation of collagen matrix, redox signaling in angiogenesis, and removal of superoxide. Some of these processes are mediated by copper-dependent enzymes that are ubiquitously or broadly expressed in various cells and tissues; others are more specialized and contribute to unique functional characteristics of specific cell types. The pigment producing tyrosinase in melanocytes, dopamine β-hydroxylase in secretory granules of adrenal gland and noradrenergic neurons of locus coeruleus, and ceruloplasmin in hepatocytes are examples of such more specialized cuproenzymes. Given a fairly limited amount of copper that organisms receive with the diet, cells face a complex and challenging task of distributing copper to various intracellular destinations to ensure activity of all proteins that require copper for their function. Inadequate copper supply results in numerous metabolic defects, including abnormal redox chemistry within a cell [1]. Lower copper levels and diminished activity of copper-dependent enzymes were reported for several neurodegenerative disorders, including Menkes disease, Alzheimer’s, and Parkinson’s disease; extreme copper deficiency is fatal.

Copper excess is equally detrimental. Redox activity of elevated copper can result in an oxidative degradation of proteins, DNA, and lipids. Copper also can change structure and activity of various metalloproteins by competing with their native metals for binding sites [2,3]. Copper overload shifts balance between the reduced and oxidized forms of glutathione (GSH and GSSG, respectively) making cellular environment more oxidizing and negatively impacting many cellular processes that rely on a precise GSH:GSSG balance. In humans, the extent of damage associated with elevated copper is exemplified by liver failure and neurodegeneration in patients with Wilson’s disease, a genetic disorder caused by mutations in a copper transporting ATPase ATP7B and copper overload [4]. To balance and distribute copper, cells evolve a complex system of transporters and small copper shuttles. Among them, Atox1 has emerged as an important “copper-router” that is centrally positioned to influence copper distribution, especially when cellular redox environment changes. 

Atox1 is a small cytosolic protein of only 68-amino acid residues that is conserved among different phyla and has 85% sequence identity in mammalian species. In human cells, Atox1 shuttles copper to the secretory pathway where it transfers copper to the copper transporting ATPases ATP7A and ATP7B located in the *trans*-Golgi network and various endocytic vesicles. This process enables maturation of copper-dependent enzymes within the secretory pathway and also maintains copper levels in a cytosol and mitochondria. Extensive genetic and biochemical studies provided strong support for the primary function for Atox1 as a copper chaperone, i.e., a protein which binds and restricts copper reactivity, while carrying copper to specific destinations within a cell. Intriguingly, Atox1 was originally identified in *Saccharomyces cerevisiae* as a molecule capable of antioxidant defense; hence its name (Atx1 in yeast) [5]. Mammalian Atox1 was also shown to protect cells against hydrogen peroxide-induced oxidative damage [6]; and inactivation of Atox1 greatly increases cell sensitivity to glutathione depletion and other stressors [7]. It was established that the copper-binding site of Atox1 is sensitive to cytosolic redox environment [7,8] and that changes in the Atox1 levels may contribute to inflammatory response and antioxidant defense [9,10]. Altogether, these observations suggest the role for Atox1 in a cellular redox balance or response to oxidative stress, but up to this day, it remains unclear whether antioxidant role is a derivative of the Atox1 copper-shuttling function or these two activities are mechanistically distinct. In this review, we summarize available experimental data that suggest that the two Atox1 functions might be coupled.

## 2. Atox1 as a Copper Chaperone

### 2.1. Copper Chaperones

Among biologically essential metals, copper is particularly important as a cofactor of various oxidoreductases. To limit reactivity of copper before it is incorporated into appropriate enzymes, cells sequester copper using predominantly thiol-containing proteins and peptides. The most abundant thiol-containing molecule—glutathione—is present at millimolar concentrations. Glutathione facilitates copper entry into cells [11] presumably by facilitating copper dissociation from a high affinity copper transporter CTR1. Copper binds to glutathione (*K_d_* = 9 × 10^−12^ M) [12] via Cu-thiolate interaction and the Cu-GSH complex can mediate copper transfer to some proteins in a cell [13], although this role is mostly carried out by specialized proteins. These copper carriers use a thiolate group of Cys residues and thioester in Met residues to form copper binding sites. Compared to the binding sites for redox-silent metals such as Na^+^, K^+^, Ca^2+^, Mg^2+^, or Cl^−^ (which have a typical *K_d_* in submicromolar to millimolar range [14,15]), affinities of copper binding sites are markedly higher ranging from femto- to zeptomolar [16]. Consequently, intracellular copper is most likely never free. In yeast, amount of free copper in the cytosol was estimated to be less than 1 atom per cell [17], which ensures secure redox environment.

**Figure 1:**
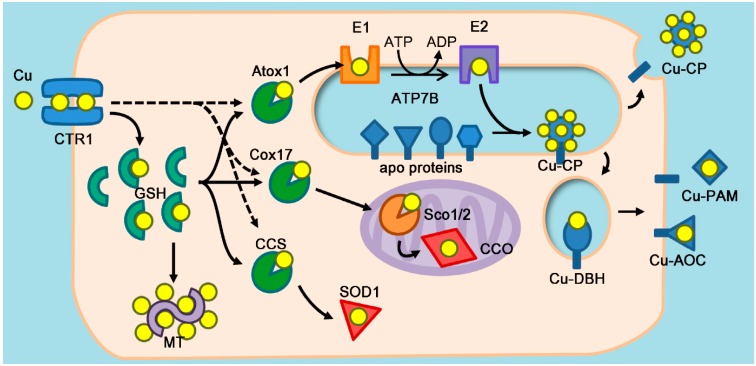
**Major routes of intracellular copper trafficking.** Copper (yellow spheres) enters the cell via the copper importer CTR1, located at the plasma membrane, and binds to thiol metabolites including glutathione (GSH). At least three different cytosolic copper chaperones (Atox1, Cox17, CCS) compete for Cu-GSH pool and sort Cu to specific destinations. Excess copper is stored as a Cu-metallothionein (MT) complex. Arrows represent the routes of intracellular copper trafficking. Alternative routes (direct transfer from CTR1 to copper chaperones) are indicated by dashed arrows. Cu-Atox1 transfers copper to the copper transporting ATPases (ATP7A and ATP7B) located in the membranes of trans-Golgi network (TGN) and secretory vesicles. ATP7B undergoes ATP-dependent conformational change from a Cu-bound state (E1) into a low affinity state (E2). Copper dissociates into lumen where it is incorporated into various copper-dependent enzymes including ceruloplasmin (CP), dopamine β-hydroxylase (DBH), peptidylglycine α-amidating monooxygenase (PAM), and other oxidoreductases.

While bound to thiol ligands, copper is shuttled to various intracellular destinations, i.e., copper-dependent enzymes in the cytosol, mitochondria, and the lumen of vesicular compartments [18]. Copper is then transferred between the dedicated metal carrier protein and its target(s) via specific protein-protein interactions. Figure 1 illustrates current view of chaperone-mediated copper sorting to different destinations within a cell. While the main players and events in intracellular copper distribution have been firmly established, specific steps are yet to be fully understood. The mechanism of copper entry into the cytosol has been a matter of debate. It is clear that a high affinity copper transporter CTR1 is responsible for the majority (about 70%) of copper entering the cell. The identity of molecules that mediate residual copper uptake remains unknown. It has been recently proposed that copper can be retrieved from CTR1 by cytosolic proteins (copper chaperones), which dock to the plasma membrane in the vicinity of CTR1 [19,20]. However, downregulation of two major copper chaperones (CCS and Atox1) has no effect on the rate of copper entry indicating that the direct transfer of copper from CTR1 to chaperones, if it occurs, cannot be a rate limiting step of copper uptake [11]. In contrast, glutathione depletion significantly diminishes the rate of copper uptake [11] and therefore glutathione may serve as an initial “weak” ligand, from which then copper is retrieved by the chaperones, which have an order of magnitude higher affinity for copper [16].

Copper chaperones are known to deliver copper in the cytosol, to the secretory pathway and mitochondria. Atox1 is the first discovered copper chaperone; it supplies copper to the secretory pathway. Mitochondria require copper for maturation of cytochrome c oxidase (CCO), which catalyzes the terminal reaction of the respiratory chain and is embedded in the mitochondrial inner membrane. Copper delivery to CCO is facilitated by concerted actions of copper chaperones Cox17, Cox11, and Sco1/2. Cox17 is a soluble metallochaperone that is found in both cytosol and the intermembrane space of mitochondria (IMS), whereas Sco1 and Sco2 are homologous membrane proteins with a thioredoxin-like domain facing IMS. Recent data suggest that Sco1 receives copper from Cox17 in the reaction coupled to electron transfer [21] and then delivers copper to CCO via specific protein–protein interactions. Sco2 also receives copper from Cox17 but acts as an oxidoreductase, reducing the metal coordinating Cys residues of CCO. Interestingly, copper binding to Sco2 greatly facilitates the redox reaction [22]. A somewhat similar phenomenon is observed for CCS, the copper chaperone for cytosolic superoxide dismutase (SOD1). SOD1 receives its copper cofactor predominantly from CCS, although in human cells glutathione can substitute for CCS [13]. CCS also catalyzes the formation of an intramolecular disulfide bond in SOD1 through a thiol-disulfide exchange [23]. This reaction can occur in the absence of bound copper, but copper binding to SOD1 facilities formation of the disulfide bond. The soluble copper chaperones (CCS, Atox1, Cox17, and possibly others) compete for copper in the cytosolic pool to facilitate copper supply to their specific target compartments. In a case of cytosol-mitochondria copper shuttles, copper transfer involves a redox reaction. Recent data suggest that cellular redox status may play an important role in a copper-chaperone function of Atox1.

**Figure 2:**
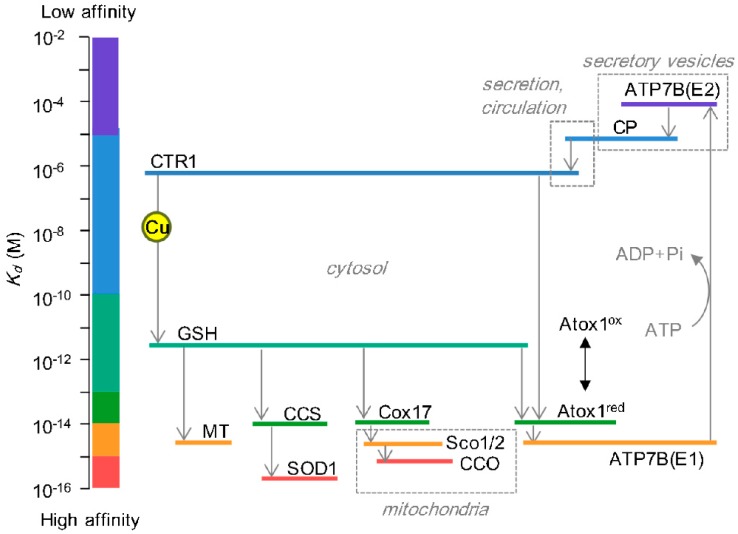
**Copper affinities of proteins involved in intracellular copper trafficking.** Movement of copper is represented by *gray lines*. Copper is transferred from one protein to another with higher copper affinity. Affinity values are based on [12] (Atox1, Cox17, ATP7B, CCS, Sco1/2, cytochrome c oxidase, SOD1, MT, GSH) and [28] (C-terminus of CTR1). CP has one high affinity site (*K_d_* = 10^−7^ M) and six low affinity sites (*K_d_* = 10^−5^ M) [29]. Copper affinity of ATP7B changes upon ATP-dependent conformational transitions; the high affinity E1 state is accessible from the cytosol and a low affinity E2 state is open to lumen). *Dashed lines* represent compartments physically separated from the cytosol.

The driving force of the directional copper trafficking to each destination is the gradient of increasing copper-binding affinities [12]; copper is transferred from the lower to higher affinity sites (Figure 2). In the mitochondrial route, copper is relayed through the copper importer CTR1 (*K_d_* > 3 × 10^−6^ M), glutathione (*K_d_* = 9 × 10^−12^ M), copper chaperone Cox17 (*K_d_* = 2×10^−14^ M), and the acceptor protein CCO (*K_d_* = 7×10^−16^ M) [12]. The cytosolic route also follows the gradient of copper affinities. In contrast, some copper acceptors within the secretory pathway have moderate copper binding affinities even lower than Atox1 (*K_d_* = 2 × 10^−14^ M). For example, dissociation of copper ions from tyrosinase in the lumen may occur so copper needs to be re-supplied [24]. Copper is also secreted as a complex with a small (<3 kDa) copper carrier (SCC), the molecular identity of which remains unknown. Considering that SCC can transfer copper to CTR1 [25], copper binding affinity of SCC is expected to be comparable or lower than that of CTR1. Thus, copper binding affinities of acceptor proteins/metabolites in the lumen of the secretory pathway are often comparable/lower compared to proteins in a cytosol or the transporter, raising a question “what is the driving force for copper transfer?” The answer may be found in the ATP-dependence and pH-dependence of this pathway. ATP7A and ATP7B are thought to undergo an ATP-dependent conformational change between the inward(cytosol)-open E1 state and outward(lumen)-open E2 state [26]. In E1 state, the protein forms an high affinity copper binding site(s) in the transmembrane region, facilitating copper transfer from Atox1. Upon ATP hydrolysis, the protein isomerizes to E2 state in which copper binding site(s) is rearranged, presumably to lower affinity, and also faces the lumen of compartment with lower pH that facilitates copper release [27]. The release of copper from the transporters triggers further conformational changes that block copper re-binding thus enabling transfer and activation of the downstream Cu-proteins/carriers.

### 2.2. The Structural Basis of the Copper Chaperone Function of Atox1

**Figure 3:**
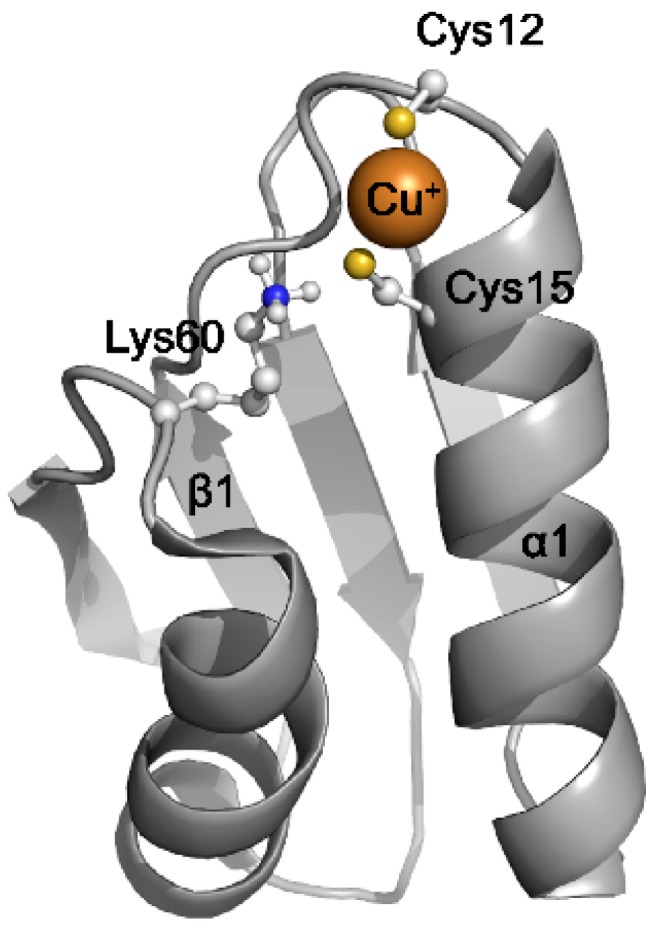
**Structure of human Atox1.** Solution structure of human Atox1 is represented as a cartoon model [PDB code 1TL4] [35]. The copper binding Cys residues and the conserved Lys residue are shown.

Human Atox1 is a 7.4 kDa soluble protein folded into a ferredoxin-like βαββαβ structure [30] (Figure 3). Anti-parallel β sheet forms the backbone of the molecule. Two short α helices “resting” at the β sheet contain two functional Cys residues as commonly observed for ferredoxin-like small oxidereductases including glutaredoxins and thioredoxins. In Atox1, the sequence motif MxCxxC on β1-α1 coil forms a conserved copper binding site to which one Cu^+^ atom binds with a dissociation constant *K_d_* of 2 × 10^−14^ M [31]. Although Met is conserved, it does not provide ligands for copper coordination, which is mediated by two Cys residues. The conformational difference between the apo-Atox1 and Cu-Atox1 is subtle and involves increased rigidity of the β1-α1 coil upon copper binding [32]. The surface of the two α helices are positively charged and these positive residues are also required for copper chaperone function [33] and interaction with the metal binding domain of ATP7A and ATP7B [34]. Recent structural analysis [19] revealed that Atox1 can bind to the lipid bilayer through electrostatic interaction of the positive patch between α1α2 and lipid head groups. Mutations of positively charged amino acids in the patch disrupt lipid-Atox1 interaction in vitro and decreased the amount of Cu-Atox1 form in cells, suggesting the role of positive patch in copper acquisition at the membrane or retention [19]. In cells, Atox1 may also acquire copper from Cu-glutathione complex. Glutathione, which is present at millimolar concentrations, serves as a stabilizing buffer for an exchangeable reduced copper. Given known abundance of Atox1 and glutathione as well as the affinities of respective copper complexes, even submillimolar concentrations of copper can be effectively chelated.

The mechanism of copper transfer from Atox1 to human ATP7A and ATP7B remains sketchy. Strong experimental evidence points to direct interactions between Atox1 and the N-terminal copper binding domain (NBD) of ATP7A/B as well as the transfer of copper from Atox1 to various metal binding sites in NBD [36,37]. Consistent with these results, the ATPase activity of ATP7B was shown to be stimulated by Cu-Atox1 but not apo-Atox1 [38]. Studies of bacterial copper transporting ATPases revealed that in the absence of NBD, the intramembrane copper transport site can still receive metal from the chaperone [39]. The atomic model of bacterial copper transporting ATPase (*Legionella pneumophila*, PDB code 4BBJ, 38% identity with human ATP7B) highlighted the positively charged cytosolic surface near the first and second trans-membrane segments, which seemingly provides a dock site for Legionella CopZ, which has an Atox1-like domain [40]. In the proposed model, copper dissociates from the chaperone and enters the vestibule approaching the transport site. The invariant nature of the proposed “entry site” makes this model appealing. However, driving forces for copper release and entry into the transporter remain unclear. Further direct experimental data are also needed, especially for human pumps, to identify the sequence of events during copper delivery and the role of Atox1 at each step of this process.

### 2.3. Enzymes that Require Atox1 Function

By transferring copper to ATP7A/B, Atox1 assists metalation of the downstream copper-dependent enzymes located in the lumen of secretory pathway. The best known example of the role of Atox1 in maturation of cuproenzymes is a yeast copper dependent ferroxidase Fet3p. Fet3p receives copper within the secretory pathway from the copper transporting ATPase Ccc2p (the yeast orthologue of ATP7A/B) and catalyzes Fe^2+^→Fe^3+^ conversion to facilitate iron uptake through Fe^3+^ permease, Ftr1p [41]. Deletion of Ccc2p decreases ferroxidase activity causing iron deficiency, which can be rescued by copper supplementation. Deletion of Atx1, yeast homologue of Atox1, phenocopied *∆Ccc2p*, providing the first evidence for copper chaperone role of Atx1 [42]. Importance of Atx1 in Fet3p activation is further illustrated by upregulation of genes encoding these proteins by the same trans activator Aft1p [42] and similar response to iron starvation [5]. In humans, a homologous Cu-ferroxidase ceruloplasmin (CP) is synthesized in hepatocytes and secreted into plasma where it generates Fe^3+^ to facilitate iron loading to transferrin.

Many other essential copper-dependent enzymes are metallated in the secretory pathway, including peptidyl-alpha monooxygenase (PAM), which is involved in peptide amidation and is indispensable for life [43]. Lysyl oxidases (LOXs) catalyze oxidative stabilization of extracellular collagen by cross-linking lysine residues. Insufficient copper supply to LOXs results in loss of skin elasticity and osteoporosis, as seen in Menkes disease and ATP7A^−/−^ and Atox1^−/−^ mice. It was recently demonstrated that copper chaperone function of Atox1 is required for vascular endothelial growth factor (VEGF) -induced angiogenesis via LOX activation [9]. Mice lacking Atox1 showed suppressed neointima formation after vascular injury, which is accompanied by the decreased accumulation of vascular smooth muscle cells within neointima [44]. SOD3 is a copper-dependent superoxide dismutase that matures within the secretory pathway and functions on the cell surface. It is also secreted from the endothelial cells into the blood and regulates angiogenesis through redox signaling [45]. Atox1^−/−^ mice have lower SOD3 activity partly due to low efficiency of copper loading to the protein [46]. Attenuated production as well as specific activity of SOD3 contributes to the exaggerated hypertensive response to angiotensin II in Atox1^−/−^ mice [46]. Tyrosinase is a copper-dependent oxidase involved in the production of melanin in melanosome. Melanosome pigmentation requires continuous copper supply from the cytosol and this process is mediated by Atox1 and ATP7A [24]. Collectively, multiple biological activities depend on copper delivery to the secretory vesicles/TGN and this flow is critically dependent on the copper chaperone function of Atox1.

### 2.4. Atox1 as a Calibrator of Cellular Copper Load

To maintain cellular copper load in a permissible range, the rates of copper absorption, utilization, and excretion must be balanced both at the organismal and cellular levels. The secretory pathway is considered to be the major route of cellular copper efflux and impairment of this route leads to abnormal elevation of cellular copper level and excessive copper entry into nucleus, mitochondria and, possibly, other compartments. In enterocytes, the copper efflux ATPase ATP7A is highly expressed enabling efficient entry of dietary copper into a bloodstream. This process is severely affected by mutations in ATP7A gene (Menkes disease), resulting in copper accumulation in intestine. ATP7B exports copper from the liver into the bile. Similarly, mutations in ATP7B (Wilson’s disease) affect biliary copper excretion, resulting in hepatic copper accumulation, which may trigger liver failure if not treated. Thus, copper sorting to the secretory pathway critically determines the cellular copper load and needs to be finely calibrated depending on the amount of copper absorbed/utilized within a cell.

Atox1 facilitates copper shuttling toward the secretory pathway and, together with ATP7A and ATP7B, plays a pivotal role in controlling cellular copper load and distribution [47]. Consistent with this role, Atox1^−/−^ mouse embryonic fibroblasts (MEF) show abnormal elevation of intracellular copper [48]. In these cells, ATP7A is constitutively relocalized from TGN to vesicular structures, indicative of cytosolic copper accumulation [49], which also seems to suggest that the rate of copper uptake into a cell exceeds the rate of copper export. Using fluorescent copper sensor, cytosolic copper accumulation was visually confirmed in cells treated with Atox1 siRNA [50]. The loss of Atox1 and ATP7A/B function not only depletes secretory pathway of copper but has important consequences for other intracellular compartments. Loss of ATP7B function in ATP7B^−/−^ mice was associated with an approximately 100-fold increase of nuclear copper concentration, demonstrating significance of the secretory pathway in preventing nuclear copper overload [51]. The form of copper entering and retained in the nuclei is not entirely clear. Atox1 has ability to transiently enter the nuclei and affect cellular transcriptional responses [52], however at steady state the primary location of Atox1 is in the cytosol [7]. 

It was also shown that different cellular compartments can compete for the limited pool of bioavailable copper in the cytosol. Hypoxia-mediated inactivation of CCS, a copper chaperone for cytosolic SOD1, redirects intracellular copper flow from the cytosol toward the secretory pathway, producing less activity of cytosolic SOD1 and higher amounts of ceruloplasmin in the secretory pathway [53]. Function of Sco1, a mitochondrial copper chaperone for CCO, is regulated by Cys oxidation and its reduced/oxidized ratio was shown to be inversely associated with the cellular copper content, suggesting the possibility that mitochondrial copper metabolism impacts on the rate of cellular copper secretion [54]. Thus change in one route of intracellular copper trafficking affects another, pointing to cross-talk between different copper routes. In this regard, the impact of Atox1 activation/inactivation may not be limited to the secretory pathway.

## 3. Antioxidant Role of Atox1

### 3.1. Atox1 Contributes to an Antioxidant Defense

The antioxidant function was first described for ATX1, the yeast orthologue of human Atox1, Overexpression of ATX1 suppressed aerobic Met/Lys auxotrophic phenotype of a strain lacking Cu/Zn-dependent superoxide dismutase SOD1 [5]. Superoxide dismutase detoxifies reactive oxygen species (ROS) endogenously produced through aerobic metabolism. SOD1 deficiency causes severe oxidative damage in multiple cellular processes including lysine synthesis [55]. Overexpression of ATX1 not only rescues the retarded growth of *sod1*^−/−^ strain, but also increased cell resistance to paraquat, a potent chemical inducer of ROS in a cell [5]. The antioxidant activity of ATX1 was observed even in the absence of SOD1 and SOD2, suggesting that ATX1 contributes to antioxidant defense independent of SOD1 and SOD2 although this effect required cellular intake of copper. 

Similarly, overexpression of mammalian Atox1 protected neuronal cells against treatment with hydrogen peroxide by reducing cellular ROS levels [6]. Under conditions of serum deprivation, which induces oxidative stress, Atox1 overexpression in neurons increases cell viability (neuroblastoma cells, teratocarcinoma cells, and hypothalamic neuronal cells) [6]. Reciprocally, Atox1^−/−^ cells are more susceptible to oxidative stress. Glutathione depletion results in the loss of Atox1^−/−^ MEFs viability, whereas the wild-type MEFs under the same conditions survive [7]. In the screening for small molecules specifically inhibiting copper chaperone functions of Atox1 and CCS, the compound DC_AC50 specifically bound to Atox1 and CCS, imposed oxidative stress, and attenuated cell proliferation, highlighting these copper chaperones as new targets in anticancer therapies [56]. In lung carcinoma cells, knockdown of Atox1 suppressed copper-stimulated cell proliferation [57]. Downregulation of Atox1 or CCS led to a significant increase in cellular ROS levels, accompanied by an oxidative change of glutathione balance [56].

The role of Atox1 in antioxidant defense is also evident from its transcriptional regulation. Cells responding to oxidative insults by upregulating antioxidant genes and Atox1 is one of such oxidant-responsive genes. In the neuronal senescence model (induced by treatment with d-galactose) accumulation of ROS was associated with increased expression of several known antioxidant genes including SOD1, glutathione peroxidase, glutathione synthase, as well as Atox1 [58]. Similarly, in the animal ischemia model, transduction of Atox1 decreased activation of astrocytes and microglia as well as lipid peroxidation in the hippocampus after ischemic insult, indicating that transduced Atox1 protects against oxidative stress-induced neuronal damage in ischemia [59]. Myeloproliferative neoplasms accumulate ROS and upregulate antioxidant genes including glutathione peroxidase, peroxiredoxin, selenoprotein P, and Atox1 [10]. These data together suggested the importance of Atox1 for combating oxidative stress. Atox1 may act directly or as a modulator of other proteins. Atox1 protected against the toxicity of amyloid β peptide and this action involved interaction with the peptidyl-prolyl-isomerase domain of immunophilin FKBP52 [60]. XRCC5, a component of DNA repairing Ku complex, was also reported to interact with Atox1 [61]. When treated with exogenous copper, XRCC5 level directly correlated with protection against oxidative damage to DNA. Thus XRCC5 and Atox1 may work together in repairing strand break and/or quenching the deleterious redox reactivity of copper [61].

### 3.2. The Proposed Mechanisms Underlying Antioxidant Role of Atox1

While antioxidant function of Atox1 has been well documented, it remains unresolved how Atox1 contributes to cellular antioxidant defense. There are two possible scenarios. Atox1 supplies copper cofactors to copper-dependent enzymes within the secretory pathway that participate in antioxidant defense, such as SOD3. Alternatively (or in addition), Atox1 may contribute to protection against oxidative stress through the mechanism that is independent of copper chaperone function. Supporting this latter scenario, mutations of the Lys residues on the positively charged patch (α1α2 helices) were shown to perturb the copper chaperone activity of yeast ATX1 without changing the ability of the mutants to function as antioxidants [33]. Furthermore, copper supplementation can correct delivery to Fet3p in *Δatx1Δsod1* yeast strain but it does not rescue the oxygen-sensitive phenotype, demonstrating that the antioxidant function of ATX1 is separate from its function in copper delivery to the secretory pathway [33].

#### 3.2.1. The SOD-like Activity

Given the ability of overexpressed Atox1 to rescue the auxotrophic *Δsod1* phenotype, early studies examined the superoxide dismutase activity of ATX1 [62]. Purified Cu-ATX1 showed activity that was 430-fold lower than that of SOD1. Thus, it is unlikely that the endogenous ATX1 has a major impact on detoxification of superoxide, at least in yeast. However, when ATX1 is overexpressed in a cell in excess (625-fold over SOD1 [62]), the total activity can be significant. Regular SOD1 activity requires copper, and ATX1 may also utilize bound copper for catalysis. Indeed, mutations in the copper binding site of ATX1 disrupt its antioxidant activity [62]. Unlike the His-based copper binding site in SOD1, ATX1 binds copper using two Cys residues, suggesting that the catalytic mechanisms may differ significantly. 

#### 3.2.2. Transactivation of Antioxidant Genes

Extracellular SOD (SOD3, EC-SOD) requires copper as a cofactor and is present in a membrane-anchored form or secreted form in the serum [63]. SOD3 is metallated within the secretory pathway, and Atox1 is essential for activation of SOD3 [64]. In Atox1^−/−^ MEF, specific activity of SOD3 is reduced (from 2.79 U/mg to 0.06 U/mg). The activity of SOD3 can be partially rescued by the in vitro copper treatment, strongly suggesting that Atox1 facilitates copper supply to the secretory pathway in which SOD3 receives its copper [64]. In addition to these expected results, the mRNA and protein levels of SOD3 were also lower in Atox1^−/−^ cells. These observations suggested an additional role for Atox1 as a regulator of SOD3 transcription. It was further demonstrated that angiotensin II-dependent upregulation of SOD3 requires Atox1 and that genetic ablation of Atox1 exacerbated hypertension and associated ROS production [46]. SOD3 gene is upregulated upon copper treatment which is abolished in Atox1^−/−^ cells [65]. It was demonstrated that Atox1 directly binds to the promoter region (GAAAGA sequence) of SOD3 gene and this interaction depends on copper [65], establishing the transactivator function of Atox1. Studies using Atox1^−/−^ MEF identified other Atox1-regulated genes in addition to SOD3 [52,65]. The effect of Atox1 on transcription could be mediated through direct transactivation or be secondary to other Atox1-dependent processes. To distinguish between these possibilities, Atox1-DNA interaction was examined by chromatin immunoprecipitation assay, which demonstrated DNA-Atox1 binding [52]. Genes dependent on Atox1 activity include cyclin D1 (required for copper-induced cell proliferation [52]) and p47phox NADPH oxidase (required for adhesion of inflammatory cells [9]). Further studies may uncover other genes affected by Atox1 levels. In this regard, a genome wide assessment using Chip-seq could be informative for funding the new physiologic roles of Atox1. For example, recent genome-wide analysis of genetic associations with changes of systolic blood pressure identified a significant locus, near Atox1 (p  =  1.0E^−8^) [66], although the role of Atox1 in these changes remains to be demonstrated.

#### 3.2.3. Coordination of Cellular Copper Distribution

Cells employ Atox1 to direct the exchangeable copper pool towards excretion in order to maintain copper levels in a cytosol and to ensure appropriate allocation of copper to cell compartments. Accumulated copper can be detoxified by high amounts of glutathione and by increased synthesis of metallothioneins. However, the effectiveness of copper sequestration is likely to differ in different type of cells. Glutathione levels in neurons are 25 times lower than in astrocytes [67] (as low as 10s of µM) and may be overwhelmed by accumulating copper. Low glutathione may make neurons particularly vulnerable to metal misbalance, as suggested by neuronal degeneration observed in such CNS pathologies as Parkinson’s disease [68], Alzheimer’s disease [69], Menkes disease [1], and Wilson’s disease [70]. Cytotoxic effects of copper can further manifest through binding to Cys residues in oxidereductases, which have Cys-x-x-Cys and Cys-x-Cys redox-active motifs [71]. Glutaredoxin is a representative Cys-x-x-Cys reductase, and its antioxidant action is severely inhibited by copper accumulation [72]. Thus, excess copper perturbs cellular redox homeostasis through its own redox activity as well as inhibition of antioxidant enzymes, and Atox1 plays a central role in maintaining the cellular copper load in a nontoxic range. 

The antioxidant function of Atox1 could also be attributed to an indirect effect on mitochondrial copper supply. In an aerobically cultured SOD1-deficient yeast strain, ROS were shown to accumulate in mitochondria and inhibit Lys4p protein, resulting in Lys auxotrophy [55]. The ability of Atox1 to correct the Lys auxotorophy strongly suggests that Atox1 can suppress ROS generation in mitochondria.

## 4. Redox Regulation of Atox1

The thiol-based redox signaling describes cellular events that involve the post-translational reduction or oxidation of sulfur moieties in protein cysteines. This redox signaling has emerged as a mechanism through which various important cellular processes are regulated. Recent studies suggest that copper distribution within the cell depends on changes in the cellular redox status. Atox1 plays a pivotal role in “decoding the cellular redox status” into appropriate adjustment of copper homeostasis.

### 4.1. The Biochemical Basis of Atox1 Redox Properties

**Figure 4:**
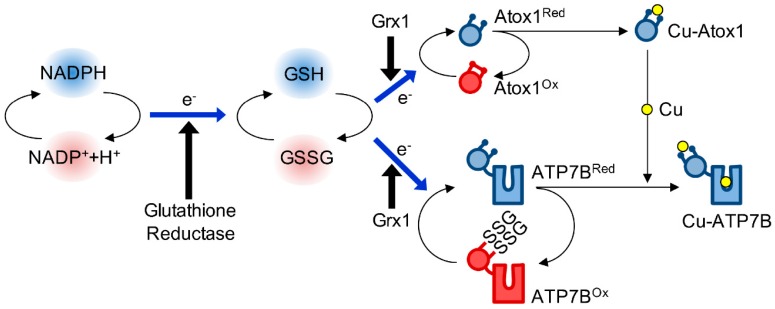
**Glutathione-dependent regulation of Atox1 and ATP7A/B**. The redox state of the chaperone and the transporters depends on glutathione system. Glutathione pair is reduced by NADPH and catalyzes reductive disulfide break in Atox1 in a reaction catalyzed by Grx1 [7,16]. Grx1 also can regulate glutathionylation of ATP7A/B [73].

Similarly to the Cys-x-x-Cys containing oxidereductases, Atox1 is highly susceptible to aerobic oxidation. Atox1 has three cysteine residues. Cys12 and Cys15 in the copper-binding Cys-Gly-Gly-Cys motif can be oxidized and form a reversible disulfide bond. A standard redox potential (*E*_0_) of −233 mV calculated for Atox1 cysteines is similar or lower than that of known oxidereductases [7]. Redox equilibration between Atox1 and glutathione pair is relatively slow (*t*_½_ = 124 min in the presence of 1 mM reduced glutathione), but is greatly accelerated by glutaredoxin 1 (Grx1) (*t*_½_ = 2.9 min). This observation suggests that, in cells, redox modulation of Atox1 depends on glutaredoxins. In agreement with this conclusion, genetic ablation of Grx1 is associated with higher copper retention in MEF cells [72]. Grx1 may also be involved in the redox modulation of ATP7A [73,74] and SOD1 [75], highlighting its importance in copper and redox homeostasis. Figure 4 summarizes current information regarding the glutathione-dependent redox regulation of Atox1 and ATP7A/B.

The structural basis of Atox1 oxidation was elucidated by Xiao and colleagues. When reacting with glutathione, Atox1 only formed an internal disulfide bond between Cys12 and Cys15. Neither glutathionylated species nor the dimer form were detected by ESI-MS [3] even in the presence of 25 mM glutathione, indicating a strong preference for formation of internal disulfides. Consequently, it was proposed that glutathionylated-Atox1 may be a short-lived intermediate, which is only transiently formed during Atox1 oxidation. 

**Figure 5:**
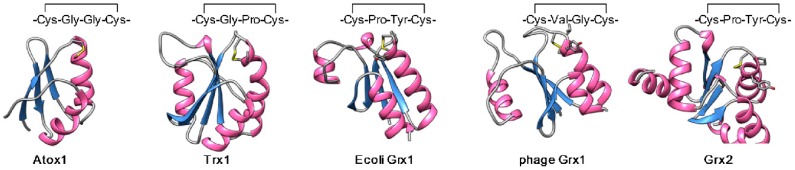
**The structures of the oxidized Atox1 and Cys-x-x-Cys reductases.** Protein backbones are rendered by ribbon and arrows. Residues in Cys-x-x-Cys motifs are shown in sticks. The structure of human Atox1 (oxidized form) was generated from the reduced apo form (PDB code 1TL5). The models of Cys-x-x-Cys reductases are human thioredoxin in oxidized state (Trx, PDB code 5DQY), *Escherichia coli* glutaredoxin in oxidized state (E. coli Grx, PDB code 1EGO), Bacteriophage T4 glutaredoxin in oxidized state (phage Grx, PDB code 1DE1), human glutaredoxin 2 in oxidized state (Grx2, PDB code 3D4M).

Conformational difference between the reduced and oxidized Atox1 is not clear because the structure for oxidized protein is not available. In the atomic model of reduced apo-Atox1 (PDB code 1TL5), positions of Cys12 and Cys15 seem favorable for disulfide formation. In a simulated conformation of oxidized Atox1, C^β^-S^γ^ of Cys12 extends in parallel with α1 helix so that S^γ^ atoms of Cys12 and Cys15 are positioned 2 Å apart. This local geometry around a disulfide bond can be observed with Cys-x-x-Cys containing reductases including thioredoxins and glutaredoxins (Figure 5). In this regard, Atox1 closely resembles members of the ferredoxin-like reductase family. In the copper-bound Atox1 (PDB code 1TL4), S^γ^ atoms of Cys12 and Cys15 are >4 Å apart, forming a space large enough for copper coordination. Upon disulfide formation, the distance between these sulfur atoms becomes too small to allow copper binding. Based on these models, it is apparent that (i) apo-Atox1 is structurally in favor of disulfide formation, (ii) redox modulation of Atox1 involves a minor conformational change, (iii) the copper binding site is disrupted by disulfide formation.

### 4.2. The Redox Status of Atox1 in Cells

In cells, the redox status of Atox1 is maintained by a glutathione pair (Figure 4). This was experimentally validated using cultured mammalian cells (HEK293). Oxidation of the Atox1 CxxC site was drastically increased in cells treated with BCNU (1,3-bis(2-chloroethyl)-1-nitrosourea, carmustine), an inhibitor of glutathione reductase [7]. Glutathione oxidation eventually impaired copper delivery to CP. As described above, the copper chaperones for mitochondria are also redox-modulated depending on mitochondrial glutathione balance [76]. Thus, glutathione pair is emerging as a common regulator of intracellular copper trafficking.

Considering that the redox status of Atox1 is controlled by glutathione pair, it is important to understand how much the redox balance of glutathione pair changes in physiologic contexts. In HEK293 cells, Atox1 is present mostly in a reduced state, consistent with the steady-state redox potential of glutathione pair −280 mV [8]), which is significantly lower than *E*_0_ of Atox1 (−233 mV [7]). It is generally assumed that the cytosol is a highly reduced environment compared to the extracellular milieu as evident from the redox potential of NADPH/NADP^+^ pair (−415 mV [77]). Although reduction of glutathione pair depends on NADPH, redox potential of glutathione pair is not the same as NADPH/NADP^+^ pair, ranging from −258 mV to −220 mV (cultured enterocytes, fibroblasts, leukemia cells) [78]. In other words, different redox pairs are not equilibrated even if they are functionally linked by various enzymes. Furthermore, glutathione is present in different cellular compartments and the glutathione pairs in these compartments can be distinctly maintained; −260 mV to −200 mV for the cytosolic glutathione and −330 mV to −280 mV for the mitochondrial pool [77]. Taking these node-to-node and compartment-to-compartment variations into account, the redox environment in the cytosol, especially the status of glutathione pair, can fluctuate. For example, drastic change in glutathione balance was observed in physiological processes such as terminal differentiation of cultured enterocytes [78,79], differentiation of ES cells to neuroprogenitors [80], serum depletion [81], or apoptosis [82]. The redox potentials determined in these studies are higher than *E*_0_ of Atox1. In other words, during these events the cytosolic environment would favor Atox1 oxidation. This prediction has been recently confirmed in studies of the redox status of Atox1 during neuronal differentiation [8]. Using a redox-sensitive GFP as an in-cell sensor of glutathione oxidation, it was demonstrated that the fraction of reduced glutathione during motor neuron differentiation became higher, which would favor Atox1 reduction. Accordingly, the degree of Atox1 oxidation was shown to change from 66% to 24%. As a result of higher percentage of reduced Atox1 (which favors metal binding), more copper was sorted to the secretory pathway. These changes allowed the differentiated motor neurons to have sufficient copper supply for the copper-containing amine oxidases in the secretory pathway (that are upregulated during differentiation), while protecting them against possible toxic effects associated with higher copper fluxes.

## 5. Conclusions

Recent studies have yielded increasing evidence for the functional link between the intracellular copper trafficking and cellular redox environment. Copper chaperone Atox1 is an important player in coupling the intracellular copper distribution to changes in the redox state of a glutathione pair in the cytosol. The copper chaperone activity of Atox1 modulates activities of copper-containing enzymes that mature in the secretory pathway. Most of these enzymes are oxidereductases, which regulate the redox-dependent reactions within and outside the cell. Consequently, Atox1 contributes to important physiological processes including inflammation, angiogenesis, iron balance, and many others. In addition, the Atox1-dependent delivery of copper to secretory pathway affects intracellular copper distribution to other compartments; loss of Atox1 alters transcription and cell proliferation. Although the underlying mechanisms of antioxidant activity of Atox1 is yet to be fully understood, the antioxidant role of Atox1 is evident from findings in various species from yeast to human. The new fluorescent probes and live cell imaging suggest that regulation of copper and redox homeostasis is a dynamic and inter-linked process.
